# Supplementary material for: Corinthian Currants Promote the Expression of Paraoxonase-1 and Enhance the Antioxidant Status in Serum and Brain of 5xFAD Mouse Model of Alzheimer’s Disease
Source: Biomolecules. 2024 Apr 1;14(4):426. doi: 10.3390/biom14040426 (PMC11047902; doi:10.3390/biom14040426)
Supplement: Supplementary file 1 [file biomolecules-14-00426-s001.zip › biomolecules-2929505-supplementary.pdf]

Corinthian currants promote the expression of paraoxonase-1 and enhance the antioxidant status in serum and the brain of the 5xFAD mouse model of Alzheimer's disease

Dimitris Lymperopoulos [1,2,†], Anastasia-Georgia Dedemadi [1,3,†], Maria-Lydia Voulgari [1,3], Eirini Georgiou [1,3], Ioannis Dafnis [1], Christina Mountaki [1], Eirini A. Panagopoulou [4], Michalis Karvelas [5], Antonia Chiou [4], Vaios T. Karathanos [4] and Angeliki Chroni [1,*]

[1] Institute of Biosciences and Applications, National Center for Scientific Research "Demokritos", Agia Paraskevi, 15341 Athens, Greece;
[2] Department of Biology, National and Kapodistrian University of Athens, Zografou, 15784 Athens, Greece
[3] Department of Chemistry, National and Kapodistrian University of Athens, Zografou, 15784 Athens, Greece
[4] Laboratory of Chemistry-Biochemistry-Physical Chemistry of Foods, Department of Dietetics and Nutrition, Harokopio University, 17676 Kallithea, Greece; chiou@hua.gr (A.C.); vkarath@hua.gr (V.T.K.)
[5] Research and Development Department, Agricultural Cooperatives' Union of Aeghion, 25100 Aeghion, Greece; mkarvelas@pesunion.gr
* Correspondence: achroni@bio.demokritos.gr; Tel.: +30-210-6503626
† These authors contributed equally to this work.

SUPPLEMENTARY FIGURES

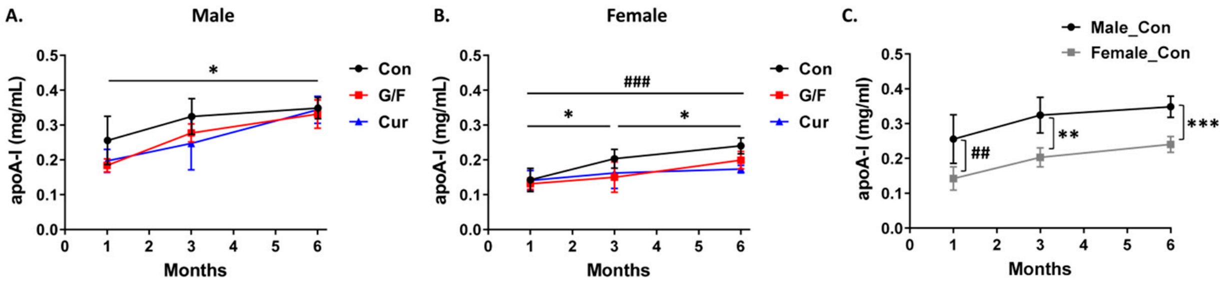

Figure S1. Serum apoA-I concentration in 5xFAD mice fed with the control diet, glucose/fructose-supplemented diet, and currant-supplemented diet. Panel (**A**) shows values for male mice, panel (**B**) shows values for female mice and panel (**C**) shows a comparison of apoA-I levels between male and female mice. Data are expressed as mean ± SD (n = 6 males, 5 females per condition). *p<0.05, ##p<0.01, **p<0.005, ###p<0.001, ***p<0.0001 for the indicated comparisons in mice fed the control diet.

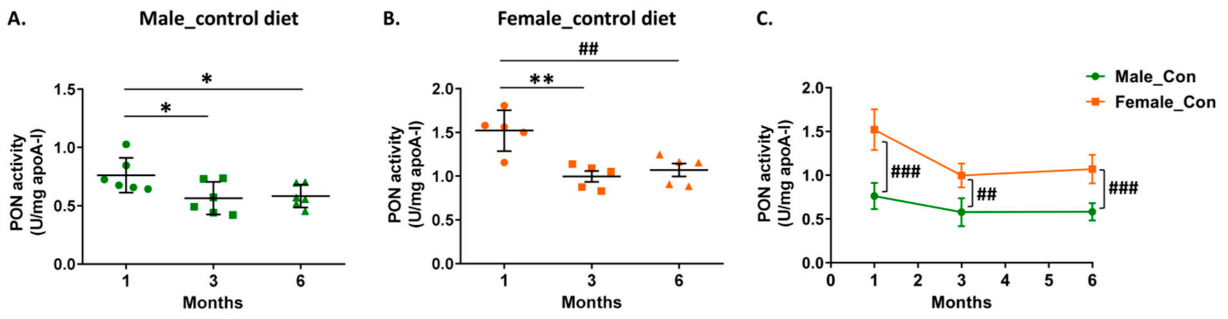

Figure S2. Effect of aging and sex on PON1 paraoxonase (PON) activity in serum of 5xFAD mice fed with the control diet. PON activity was normalized with apoA-I concentration and expressed as U/mg of apoA-I. Panel (**A**) shows values for male mice, panel (**B**) shows values for female mice and panel (**C**) shows a comparison of PON activity between male and female mice. Data are expressed as mean ± SD (*n* = 6 males, 5 females per condition). *p<0.05, ##p<0.01, **p<0.005, ###p<0.001.

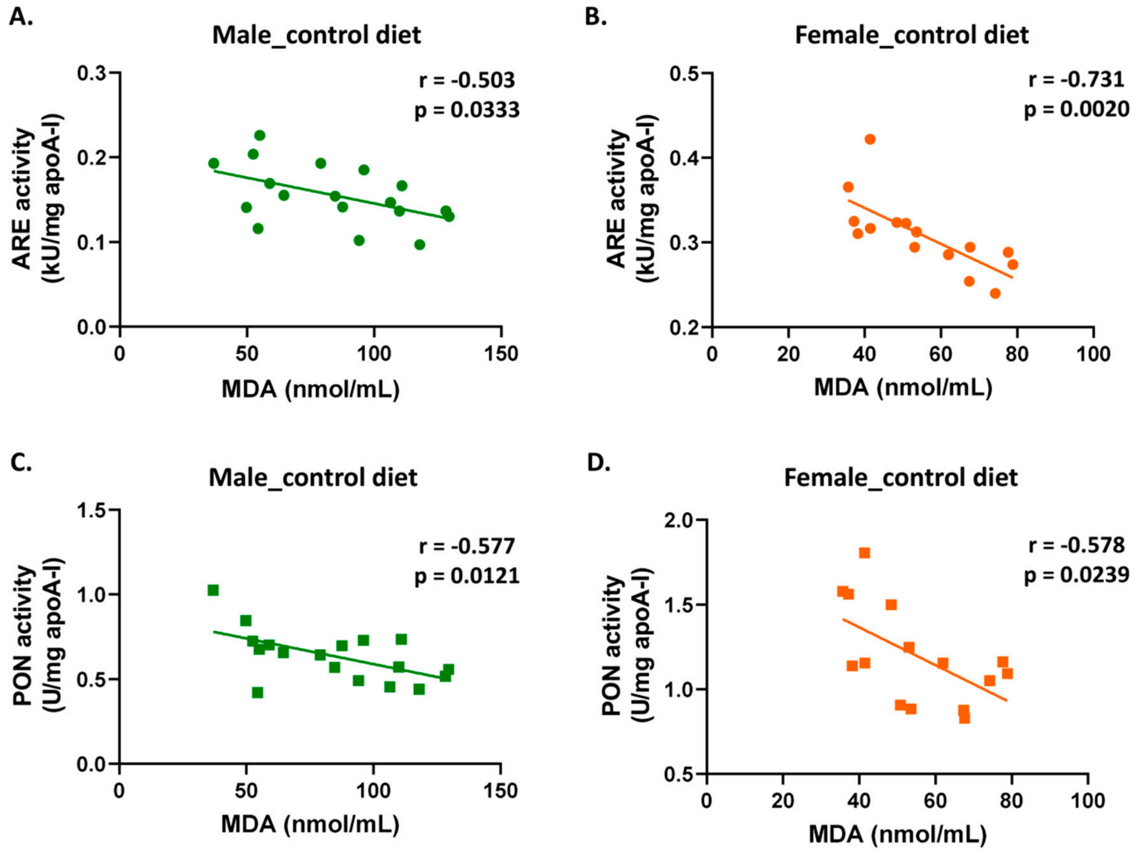

Figure S3. Correlation analysis between ARE or PON activity and MDA levels in serum of male and female 5xFAD mice fed with the control diet for 1, 3 and 6 months (control groups).

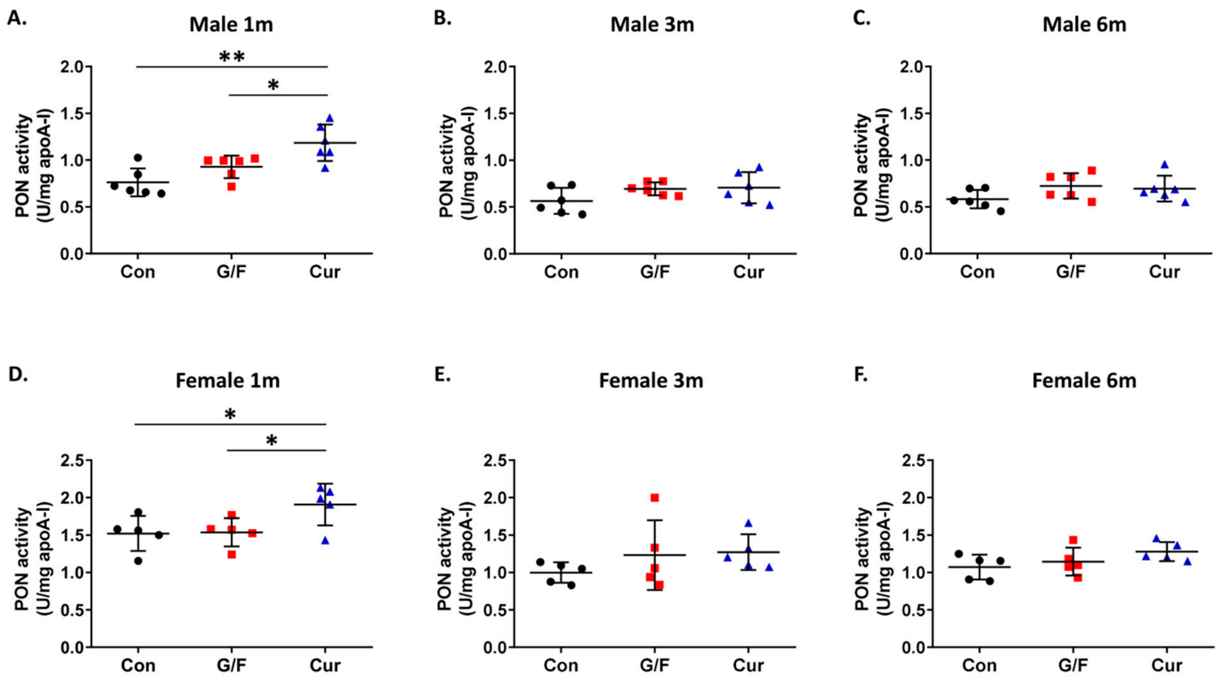

Figure S4. Time-dependent effect of currant diet on PON1 paraoxonase (PON) activity in serum of 5xFAD mice. PON activity was normalized with apoA-I concentration and expressed as U/mg of apoA-I. Panels (**A-C**) show values for male mice and panels (**D-F**) show values for female mice. Data are expressed as mean ± SD (*n* = 6 males, 5 females per condition). *p<0.05, **p<0.005.

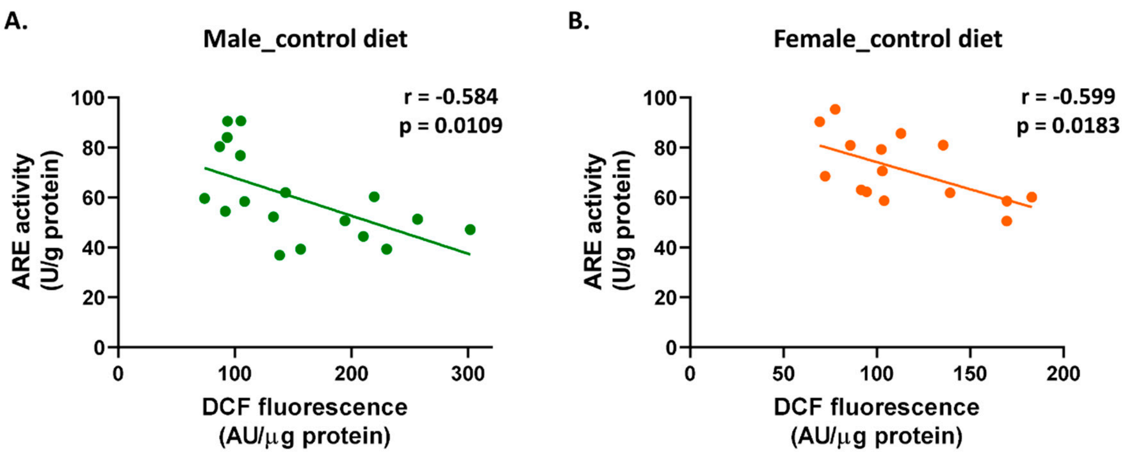

Figure S5. Correlation analysis between ARE activity and DCF fluorescence (indicative of free radical levels) in the cortical homogenate of male and female 5xFAD mice fed with the control diet for 1, 3 and 6 months (control groups).

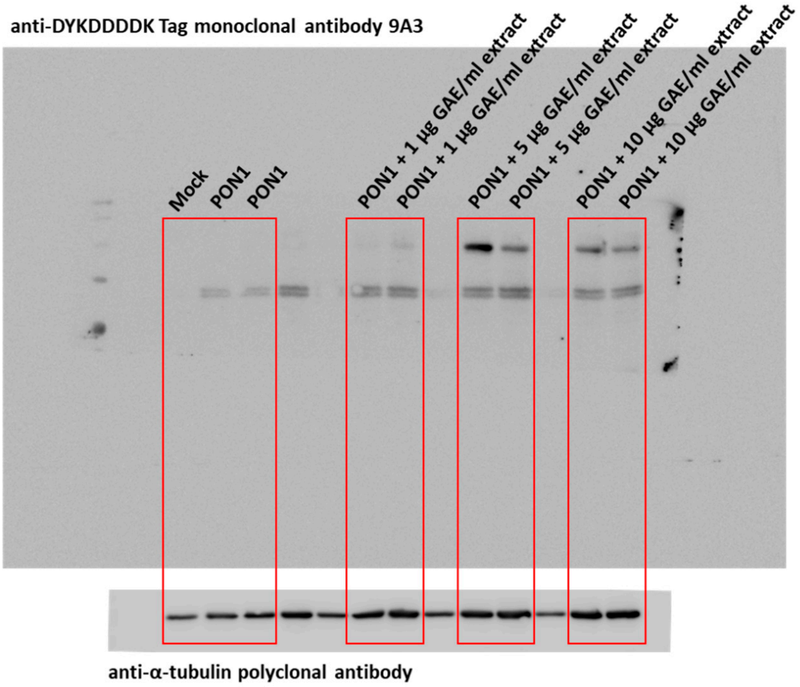

Figure S6. Uncropped immunoblots for PON1 and α-tubulin. Red frames indicate the bands presented in Figure 10D.
